# Evaluation of the Thoracolumbar Injury Classification and Severity (TLICS) Score Over a Two-Year Period at a Level One Trauma Center

**DOI:** 10.7759/cureus.43762

**Published:** 2023-08-19

**Authors:** Gabriel S Gonzales-Portillo, James C Mamaril-Davis, Katherine Riordan, Mauricio J Avila, Pedro Aguilar-Salinas, Aaron Burket, Travis Dumont

**Affiliations:** 1 Medicine, The University of Arizona College of Medicine, Tucson, USA; 2 Neurosurgery, The University of Arizona College of Medicine, Tucson, USA; 3 Neurosurgery, University of Arizona College of Medicine, Tucson, USA

**Keywords:** brace, spine orthotics, thoracolumbar, thoracolumbar injury classification and severity (tlics), spine fracture, spine trauma, spine fusion

## Abstract

Introduction

The use of the Thoracolumbar Injury Classification and Severity Score (TLICS) and other classification systems for guiding the management of traumatic spinal injuries remains controversial. TLICS is one of the few classifications that provides treatment recommendations.We sought to analyze intervention modality selection based on the TLICS scoring system.

Methods

A retrospective review of patients presenting with traumatic thoracolumbar fractures at a level 1 trauma center over a two-year period was performed. Primary endpoints for comparison analysis included visual analog scale (VAS) scores and Cobb angles during follow-up.

Results

There were 272 patients with thoracolumbar fractures, of whom 212 had TLICS of ≤3, six with TLICS of 4, and 54 with TLICS of ≥5. Of the 272 total patients, 59 were treated via surgery and 213 via non-surgical conservative methods. The VAS scores significantly decreased from presentation to last follow-up in both surgically treated and conservative groups (p<0.0001). This remained consistent in subgroup analyses of TLICS ≤ 3, TLICS = 4, and TLICS ≥ 5 (p<0.0001). Burst fractures treated conservatively had larger fracture Cobb angles versus those treated via surgery at the last follow-up, although this was not significantly associated (p=0.07). The only significant relationship with Cobb angles was in distraction fractures of the TLICS > 4 conservative group, who had significantly lower Cobb angles at the last follow-up than the TLICS > 4 surgical group (p<0.04). The "surgeon's choice" for TLICS = 4 was surgical intervention (4/6 patients, 66.7%).

Conclusion

Using the TLICS score, thoracolumbar injuries in a level 1 trauma center are more commonly TLICS ≤ 3. For patients with TLICS = 4, the surgeon's choice was most commonly surgical repair. VAS scores decreased over time from presentation between surgically and conservatively managed patients (as well as within-group analyses). The data concerning Cobb angles were more ambiguous, as larger Cobb angles in burst fractures treated conservatively did not show statistically significant differences with surgery.

## Introduction

Thoracolumbar spinal trauma is a common site of spinal cord injury and accounts for 30-60% of total spine fractures [1-4]. The thoracolumbar junction sits between the relatively immovable thoracic and mobile lumbar regions, making it prone to traumatic injury [5]. Thoracolumbar injury is classically caused by high-energy impacts, such as motor vehicle and industrial accidents [5]. With these injuries, there is a high potential for neurological deficits secondary to spinal cord injury as well as substantial healthcare costs to the individual and system at large [3-5]. Due to debilitating sequelae, proper diagnosis and treatment guidelines for thoracolumbar fractures are essential [5]. To guide such treatment protocols, prior research has been completed on the development of classification systems for thoracolumbar injuries. Different classification systems have previously been proposed, such as the ones by McAfee et al., Ferguson et al., Gertzbein et al., and Mirza et al., but these remain controversial in terms of their validity, reliability, and reproducibility [6-9]. Most of these focus on structural characteristics of thoracolumbar fractures without considering neurological complications-which are at the center of clinical decision-making for these types of injuries [6-10].

To create a more accurate and reproducible classification system, Vaccaro et al. suggested the Thoracolumbar Injury Classification and Severity score (TLICS) to facilitate communication between physicians and guide treatment decisions [11]. This simplified classification system focuses on three factors: the morphology of fracture, the posterior ligament complex integrity, and neurological status [11]. A composite score is calculated from these factors and stratifies patients into operative and non-operative groups [11]. Patients with a score of zero to three indicate conservative or non-operative management; greater than or equal to five indicates surgical management; and a score of four indicates the "surgeon's choice" for either conservative or surgical treatment [11]. Studies have shown favorable results for the reliability and validity of TLICS, as well as its utility in devising a treatment strategy and classification of the mechanism of injury [12-16]. The TLICS system unifies the language between radiologists and spine surgeons; however, it is not without shortcomings. In particular, the category of TLICS = 4, where the decision is left to the surgeon, represents a gray area. It is not infrequent to find patients in this category, and the treating surgeons are left in a conundrum to decide what is best for these patients.

In this study, we investigate the use of the TLICS treatment protocol in our level I trauma center. We aimed to analyze the TLICS score's applicability to predicting clinical outcomes in terms of pain resolution and deformity correction. The present study also sought to elucidate the cost differences between management techniques and post-TLICS scoring.

## Materials and methods

A retrospective cohort study was conducted of patients presenting with traumatic thoracolumbar fractures at our level 1 trauma emergency department at the Banner University Medical Center in Tucson, Arizona. The study period was two years between 2017 and 2018. At the time of the study, our hospital was the only level 1 trauma center in the city and had a coverage of about 1.2 million people, including the suburbs and neighboring states. Patients were identified using International Classification of Diseases Tenth Edition (ICD-10) codes, and then individual patients' charts were reviewed to remove miscoded patients. CT scans were then analyzed individually by two authors (GSGP and TMD). TLICS scores were determined by the definition described by Vaccaro et al. [11]. Each author reviewed the images, and disagreement was resolved with a discussion between reviewers. The cost associated with the trauma admission was provided by the hospital's accounting department, and this was also compared between treatment options.

Data extraction

Demographics, treatment characterizations, and outcomes were extracted from electronic medical records and placed into a customized Excel spreadsheet. Demographics included the gender of patients, ages, body mass indices, types of fractures, and vertebral levels of the fractures. Data pertaining to treatment characterization included treatment modality (surgery versus conservative), total cost of treatment, overall TLICS score, individual TLICS components, compression angle, listhesis of the vertebral body, vertebral body height loss, transitional anatomy, pars defect, mechanism of injury, and visual analog scale (VAS) scores at initial presentation. Treatment outcomes included number of surgeries; delayed surgeries (after initial trauma admission); length of hospital stay; 30-day complications; follow-up Cobb angles at 0-3 and 4+ months; and VAS scores at discharge, 0-3 months follow-up, and 4+ months follow-up. Follow-up data was also acquired from electronic medical records. 

Statistical analysis

To gather a complete retrospective cohort, individual datasets were compiled together and analyzed in Prism 8 (GraphPad, San Diego, California). Sums were aggregates of an individual category (e.g., total amount of types of fracture), and averages were represented as means (e.g., average VAS scores) with 95% confidence intervals (CI). The nonparametric student's t-tests were used for direct comparisons of data with two groups, while nonparametric one-way analyses of variance were used for data with more than two comparisons. Welch's correction was applied for all statistical relationships, which accounts for heterogeneity and variance when sample sizes vary (as was the case here). Significance was set at p<0.05.

## Results

Surgically versus conservatively treated groups

The data for 272 patients with thoracolumbar fractures were extracted, 59 of whom were treated via surgery and 213 via conservative methods. Patients treated conservatively were significantly older (mean age 54.6 years) than those who were managed with surgery (mean age 39.1 years; p<0.0001). The most common vertebral level fracture in either group was at L1: 63/213 (29.6%) and 26/59 (44.1%) of patients in the conservative and surgical cohorts, respectively. Compression fractures were more commonly seen in the conservative group (65.7%, 140/213 patients), while burst fractures were more commonly treated in the surgical group (59.3%, 35/59 patients). For the whole cohort, the most common mechanism of injury was motor vehicle accidents (54.0% of those having surgery and 54.4% of those receiving conservative measures). The second most common traumatic mechanism was a fall. Of those requiring surgery, there was a statistically significant difference between the reported height of the fall, with the patients requiring surgery falling from a higher height (mean 14 ft) compared to the conservative group (mean 5.5 ft, p=0.002). Patients treated conservatively had a significantly shorter hospital stay at a mean of 3.7 days when compared to surgical patients at a mean of 6.9 days (p<0.0001). The overall cost for conservative management ($63,332; 95% CI $47,991-$78,673) was significantly lower than that of surgery ($184,562; 95% CI $152,053-$217,071) (p<0.0001). Additional characteristics can be found in Table 1 and Table 2.

**Table 1:** Characteristics of TLICS patients stratified into surgically and conservatively treated groups TLICS - Thoracolumbar Injury Classification and Severity Scale; BMI - body mass index, T - thoracic, L - lumbar, CI - confidence interval

Characteristics of TLICS Patients	Surgery, n=50	Conservative, n=164	p-value
Gender (n, % of total)			
Male	31 (62.0%)	90 (54.9%)	0.33
Female	19 (38.0%)	74 (45.1%)	0.33
Mean age (n; 95% CI)	39.2 (50; 33.5 - 44.9)	55.5 (164; 52.4 - 58.7)	<0.0001
Mean BMI (n; 95% CI)	28.0 (46; 26.4 - 29.5)	27.4 (140; 26.4 - 28.3)	0.59
Mean total cost (n; 95% CI)	174980 (41; 150763 - 199198)	59631 (138; 43446 - 75815)	<0.0001
Type of fracture (n, % of total)			
Compression	0 (0%)	110 (67.1%)	0.59
Burst	31 (62.0%)	48 (29.3%)	0.59
Distraction	17 (34.0%)	5 (3.0%)	0.59
Translation	1 (2.0%)	0 (0%)	0.59
Multiple	1 (2.0%)	1 (0.6%)	0.59
Level of Vertebrae Fractured (n, % of total)			
T11	2 (4.0%)	14 (8.5%)	0.11
T12	15 (30.0%)	49 (29.9%)	0.11
L1	26 (52.0%)	65 (39.0%)	0.11
L2	7 (14.0%)	36 (22.0%)	0.11

**Table 2:** Outcomes of TLICS patients stratified into surgically and conservatively treated groups TLICS - Thoracolumbar Injury Classification and Severity Scale; PLC - posterior ligamentous complex integrity; MVC - motor vehicle; VAS - visual analog scale score; CI - confidence interval

Outcomes of TLICS patient	Surgery	Conservative	p-value
Median total TLICS score (n; 95% CI)	5.5 (50; 5 - 7)	1 (164; 1 - 1)	<0.0001
Median morphology of fracture score	2 (50; 2 - 4)	1 (164; 1 - 1)	<0.0001
Median neurological status score	0 (50; 0 - 2)	0 (164; 0 - 0)	<0.0001
Median integrity of PLC score	3 (50; 0 - 3)	0 (164; 2 - 3)	<0.0001
Listhesis of vertebral body (n)	4 total grade 1s	8 total grade 1s	-
Mechanism of Injury			
MVC-related (n, %))	25 (50.0%)	90 (54.9%)	0.16
Fall-related (n, %)	24 (48.0%)	68 (41.5%)	0.16
Average fall height in feet (n; 95% CI)	14.0 (20; 9.2 - 18.8)	5.1 (58; 3.2 - 7.0)	0.0002
Other (crush injury, assault) (n, %)	1 (2.0%)	6 (3.6%)	0.16
Mean kength of hospital stay in days (n; 95% CI)	6.9 (50; 5.9 - 7.8)	3.5 (164; 2.8 - 4.3)	<0.0001
Number of 30-day complications (n)	13	7	-
Mean follow-up Cobb angle (n; 95% CI)			
0-3 months	8.4 (33; 6.7 - 10.2)	8.6 (59; 7.2 - 10.0)	0.94
4+ months	8.3 (39; 6.3 - 10.2)	9.6 (31; 7.2 - 12.0)	0.45
Mean VAS score (n; 95% CI)			
Pre-op or initial presentation	8.1 (28; 7.3 - 8.9)	8.1 (106; 7.7 - 8.5)	0.94
Post-op or discharge	4.2 (32; 3.4 - 5.0)	3.8 (93; 3.4 - 4.3)	0.45
Follow-up 0-3 months	1.9 (7; -0.1 - 4.0)	2.0 (8; -0.4 - 4.4)	0.89
Follow-up 4+ months	3.7 (17; 1.6 - 5.7)	2.8 (23; 1.4 - 4.3)	0.55

Characterization of surgical versus conservative treatment stratified by TLICS

From the overall cohort, 212 had a TLICS score of <3 (eight patients treated via surgery and 204 via brace), six had a TLICS score of 4 (four patients treated via surgery and two via brace), and 54 had a TLICS score of > 5 (47 treated via surgery and seven via brace). There were 212 patients with a TLICS score of < 3, eight of whom were treated via surgery and 204 via conservative brace. There were 60 patients with a TLICS score of > 4, 51 of whom were treated via surgery and nine via conservative treatment with orthotic braces. All eight patients (100%) treated via surgery in the TLICS < 3 group had burst fractures compared to only 30% of those treated conservatively. The vast majority of fractures treated conservatively in the TLICS < 3 group were compression fractures (65.6%). The mean overall TLICS score for the surgical group was 6.3 (95% CI 5.9 - 6.7), while that of the conservative cohort was 6.0 (95% CI 5.1 - 6.9) (p=0.56). Regarding the neurological component of the TLICS, 94% of patients treated surgically had neurological deficits compared to 0% of those treated in a brace (p<0.0001). Furthermore, the mean fracture compression angle (9.8°, 95% CI 8.0 - 11.6° versus 6.0°, 95% CI 3.2 - 8.9°) and vertebral body height loss (34.1° 95% CI 28.1 - 40.2° versus 17.7° 95% CI 5.8 - 30.0°) were significantly higher in the surgical group (p=0.024 and p=0.015, respectively). Additional characteristics pertaining to patients stratified via TLICS can be found in Table 3 and Table 4.

**Table 3:** Characteristics of TLICS patients stratified by TLICS scoring TLICS - Thoracolumbar Injury Classification and Severity Scale; BMI - body mass index, T - thoracic, L - lumbar, CI - confidence interval

Characteristics of TLICS patients	TLICS <3, n=164	TLICS =4, n=5	TLICS >5, n=45	p-value
Gender (n, % of total)				
Male	89 (54.3%)	3 (60.0%)	29 (64.4%)	0.055
Female	75 (45.7%)	2 (40.0%)	16 (55.2%)	0.055
Mean age (n; 95% CI)	55.7 (164; 52.5 - 58.8)	46.6 (5; 25.2 - 68.0)	38.0 (45; 32.0 - 44.0)	< 0.0001
Mean BMI (n; 95% CI)	27.5 (139; 26.5 - 28.4)	30.2 (5; 23.6 - 36.8)	27.3 (42; 25.7 - 28.9)	0.51
Mean total cost (n; 95% CI)	62457 (136; 45889 - 79024)	119964 (5; 81760 - 158168)	166036 (38; 136156 - 195916)	< 0.0001
Type of fracture (n, % of total)				
Compression	107 (65.2%)	1 (20.0%)	0 (0%)	0.27
Burst	56 (34.1%)	4 (80.0%)	20 (44.4%)	0.27
Distraction	0 (0%)	0 (0%)	23 (51.1%)	0.27
Translation	0 (0%)	0 (0%)	1 (2.2%)	0.27
Multiple	1 (0.7%)	0 (0%)	1 (2.2%)	0.27
Level of vertebrae fractured (n, % of total)				
T11	13 (7.9%)	0 (0%)	3 (6.7%)	0.037
T12	48 (29.3%)	1 (20.0%)	15 (33.3%)	0.037
L1	69 (42.1%)	3 (60.0%)	19 (42.2%)	0.037
L2	34 (20.7%)	1 (20.0%)	8 (17.8%)	0.037
Treatment modality (n, % of total)				
Surgery	7 (4.3%)	4 (80.0%)	39 (86.7%)	0.55
Conservative	157 (95.7%)	1 (20.0%)	6 (13.3%)	0.55

**Table 4:** Outcomes of TLICS patients stratified by TLICS scoring TLICS - Thoracolumbar Injury Classification and Severity Scale; PLC - posterior ligamentous complex integrity; MVC - motor vehicle; VAS - visual analog scale score; CI - confidence interval

Outcomes of TLICS patients	TLICS <3	TLICS =4	TLICS >5	p-value
Listhesis of vertebral body (n)	8 total grade 1s	1 total grade 1	3 total grade 1s	-
Mechanism of injury				
MVC-related (n)	88 (53.7%)	2 (40.0%)	26 (57.8%)	0.15
Fall-related (n)	68 (41.5%)	3 (60.0%)	19 (42.2%)	0.15
Average height in feet (n; 95% CI)	5.9 (45; 3.6 - 8.3)	19.7 (3; -14.6 - 53.9)	11.4 (15; 6.2 - 16.7)	0.16
Other (crush injury, assault) (n)	8 (4.8%)	0	0 (0%)	0.15
Mean length of hospital stay in days (n; 95% CI)	3.7 (164; 2.9 - 4.4)	5.6 (5; 3.9 - 7.3)	6.5 (45; 5.3 - 7.6)	0.0021
Number of 30-day complications (n)	6	0	14	-
Mean follow-up Cobb angle (n; 95% CI)				
0-3 months	8.3 (62; 7.0 - 9.6)	8.0 (3; -7.1 - 23.1)	9.1 (27; 7.0 - 11.1)	0.83
4+ months	9.1 (38; 6.9 - 11.2)	11.0 (2; -27.1 - 49.1)	8.5 (30; 6.2 - 10.7)	0.78
Mean VAS score (n; 95% CI)				
Pre-op or initial presentation	8.1 (102; 7.7 - 8.5)	8.0 (5; 6.0 - 10.0)	8.1 (27; 7.2 - 9.0)	0.99
Post-op or discharge	3.7 (104; 3.2 - 4.1)	4.6 (5; 2.5 - 6.7)	4.0 (28; 3.1 - 4.9)	0.46
Follow-up 0-3 months	2.9 (55; 2.2 - 3.7)	3.3 (3; -3.8 - 10.5)	2.9 (12; 1.1 - 4.7)	0.97
Follow-up 4+ months	2.8 (22; 1.3 - 4.2)	0.3 (3; -1.1 - 1.8)	2.7 (11; 0.6 - 4.8)	0.0069

TLICS score 4 group

Of 272 total patients, six had a TLICS score of 4 (four patients treated via surgery and two via brace). TLICS = 4 patients (mean age 41.7 years; 95% CI 21.1 - 62.2) were younger than those in the TLICS < 3 group. Of the six patients, four had burst fractures. Interestingly, the "surgeon's choice" for treatment management of TLICS = 4 was surgical intervention (four out of six patients for a rate of 66.7%). The length of stay for TLICS = 4 patients was a mean of five days, which was longer than those with TLICS < 3 (mean 3.8 days) but shorter than those with TLICS > 4 (mean 6.4 days; p=0.003). Regarding VAS pain scores, the TLICS = 4 group had the lowest, as well as the greatest decrease in, VAS scores with long-term follow-up at 4+ months after treatment initiation (p=0.0006). Other characteristics and associations can be found in Table 4.

VAS score analysis

VAS scores for the surgical group showed a statistically significant improvement after surgery from admission (mean score 8.1/10) to three-month follow-up (mean 2.3/10; p≤0.0001). The same was found for the conservative group from admission (mean score 8.2/10) to three-month follow-up (mean score 2.6/10; p<0.0001). In addition, intra-group analysis via stratified TLICS score also showed significant improvement after either surgery or conservative measures from admission to three-month follow-up (Table 5).

**Table 5:** Intra-group comparisons of visual analog scale score progression from initial presentation to last follow-up visit Significance was set at p<0.05

	p-value	Welch's ANOVA test W (DFn, DFd)
Surgically treated group	<0.0001	24.34 (3.000, 26.46)
Conservatively treated group	<0.0001	100.40 (3.000, 51.79)
TLICS <3	<0.0001	114.4 (3.000, 97.51)
TLICS =4	0.0002	30.40 (3.000, 6.860)
TLICS >5	<0.0001	22.36 (3.000, 34.73)

Fracture type analysis

The Cobb angles from burst fractures in surgically and conservatively managed groups were not statistically different at 0-3 or 4+ months follow-up (p=0.75 and p=0.26, respectively) (Table 6). The angles from distraction-type fractures in surgical and conservative groups were also not statistically different at 0-3 or 4+ months follow-up (p=0.61 and p=0.94, respectively) (Table 6). When stratified by TLICS score < 3, the Cobb angles from burst fractures in surgically and conservatively managed groups were not statistically different at 0-3 or 4+ months follow-up (p=0.84 and p=0.32, respectively) (Table 6). However, when stratified by TLICS score > 4, the Cobb angles from distraction fractures in surgically managed groups were significantly higher at both the 0-3 month or 4+ months follow-up visit than the conservative groups (p=0.0003 and p=0.041, respectively) (Table 6). Not all fracture types could be compared because some groups did not have that specified fracture.

**Table 6:** Inter-group comparisons of Cobb angles by fracture type at last follow-up visit Significance was set at p<0.05; not all fracture types could be assessed as some groups did not have the respective fractures (please refer to Tables 1-2)

	p-value	Unpaired t-test with Welch's correction (F, DFn, DFd)
Burst fractures in surgically vs. conservatively treated groups (0-3 months)	0.75	1.218, 31, 22
Burst fractures in surgically vs. conservatively treated groups (4+ months)	0.26	21.125, 21, 24
Distraction fractures in surgically vs. conservatively treated groups (0-3 months)	0.61	2.304, 2, 9
Distraction fractures in surgically vs. conservatively treated groups (4+ months)	0.94	1.381, 2, 13
Burst fractures in surgically vs. conservatively treated groups when TLICS <3 (0-3 months)	0.84	1.075, 4, 31
Burst fractures in surgically vs. conservatively treated groups when TLICS <3 (4+ months)	0.32	1.296, 21, 6
Distraction fractures in surgically vs. conservatively treated groups when TLICS >4 (0-3 months)	0.0003	-
Distraction fractures in surgically vs. conservatively treated groups when TLICS >4 (4+ months)	0.041	-

## Discussion

The present study illustrates the utility of both conservative and surgical management of thoracolumbar fractures based on the TLICS scoring system. Patients treated with either surgery or conservative treatment had a statistically greater improvement of their pain, via VAS scores, compared to the initial presentation, and these results were carried to the last follow-up visit. These results held true for the TLICS = 4 patients, where they had the lowest overall and greatest decrease in VAS scores with long-term follow-up. Prior reports suggest that solely conservative therapy for TLICS score of 4 may not be enough to control pain. In a 2016 retrospective study, Pneumaticos et al. found a non-significant decrease in VAS scores of 20 patients with a TLICS score of 4 who were treated conservatively [17]. VAS scores in that study decreased only from 7.8 to 6.4 over a 28-month period [17]. This contrasts with the results here, where there was a decrease of VAS scores from 8.0 to 2.5 over three months in TLICS = 4 patients who were treated with either surgery or a brace. Further, Karaali et al. found that TLICS = 4 patients treated via surgery (74 patients) showed more improvement in VAS scores at six months follow-up when compared to patients treated non-operatively (76 patients; p<0.001) [18]. Although only conjecture from retrospective studies, there may be a more prominent role for surgery, rather than bracing, in TLICS = 4 groups when pain is the patient's limiting symptom.

The feasibility of using the TLICS system for guiding clinical treatment in terms of Cobb angle, and by extension, deformity correction, was not as practical. Only distraction fractures in the TLICS > 4 conservative group had significantly lower Cobb angles at the last follow-up. This is not clinically useful information, however, as TLICS > 4 patients with a distraction fracture treated conservatively accounted for less than 5% of the patient sample. Nevertheless, some reports in the literature may suggest otherwise. Wenjie et al. found a significant improvement in Cobb angles from index injury (25.0 + 3.9 degrees) to the last follow-up (3.1 + 1.0 degrees) after treatment of thoracolumbar fractures [19]. However, they did not stratify their sample by treatment modality (conservative versus surgery) or fracture type (e.g., burst, distraction, etc.), making the data challenging to interpret [19]. In a meta-analysis of 555 patients with a TLICS score of 4, Cobb angles were found to be lower in the surgically treated group at 6, 12, and >24 months postoperatively [20]. There was no stratification of Cobb angle corrections by fracture type, however, thereby limiting its utility to guide treatment in a clinical setting [20].

Interestingly, with a mean cost of $184,526, the surgical group had significantly higher hospital expenses than the conservative group, which amounted to a mean of $63,332. Cost-effectiveness studies remain sparse in the literature [21]. Although it may seem intuitive that conservative management would be cheaper in comparison to surgical repair for thoracolumbar fractures, the data presented here points to the stark disparity in cost, a >$120,000 gap. This is seemingly unavoidable, however, as TLICS > 5 almost exclusively requires surgery to avoid instability. Moreover, while analyzing fracture type, we found that burst fractures treated conservatively with a brace had a higher Cobb angle, and thus a higher degree of deformity, on the last follow-up versus those treated with surgery. Nonetheless, patients had improved pain compared to the initial presentation. However, our follow-up time periods ranged from 0-4+ months, and it is difficult to say if this increase in Cobb angle would have continued to demonstrate further deformity after a longer period of time.

Limitations

The present study has some limitations. As a retrospective study, this report is inherently prone to selection and recall bias, and only associations can be drawn from our analyses. Our report was also limited by including all ICD-10 coded patients on the electronic medical record and having two independent authors review CT scans. Furthermore, our patients were scored using the TLICS system. There are multiple scoring systems to classify thoracolumbar injuries, including the increasingly used AO classification system. TLICS was used at our institution as it offers treatment guidance and is less prone to inter-reader variability when compared to the AO classification [16]. Also, not all patients in our study had an MRI to evaluate the integrity of the posterior ligament complex per TLICS scoring, thereby potentially misclassifying injuries to posterior ligament complexes. Although previous studies have shown that MRI offers more detailed imaging and may alter the final TLICS score, spinal MRI is not part of routine care in many trauma centers, including our own, and the specificity of these findings has been debated. At our institution, MRIs are only obtained if neurologic deficits are present after trauma. 

We also examined patients who had varying times for follow-up, ranging from 0-4+ months. This lack of standardized follow-up makes it difficult to draw firm conclusions about the reported data. Additionally, Cobb deformity cannot be fully classified in such a short follow-up period. However, the follow-up times reported in the present study do offer an understanding of pain relief for the patient as well as the progression of bone repair and healing.

## Conclusions

While patients with compression fractures treated conservatively had worse deformities at the last follow-up, all patients, regardless of treatment option, showed improvement in pain scores at the last follow-up. There is also a stark, seemingly unavoidable disparity in cost between conservative and surgical management for thoracolumbar fractures, a gap that is greater than $120,000 in healthcare costs.
